# Mine Personnel Positioning System Based on Dual-Wavelength Phase Unwrapping

**DOI:** 10.1155/2022/9125594

**Published:** 2022-09-10

**Authors:** Hongwei Zhang, Lin Zhang, Zhixin Zhao

**Affiliations:** School of Electronic and Electrical Engineering, Lingnan Normal University, Zhanjiang 524048, Guangdong, China

## Abstract

During the construction of coal mine laneway, due to the particularity of the geographical location and structure of the laneway, its accidents often occur, causing great trouble to the personal and property safety of the construction personnel. Therefore, according to the environmental characteristics of the coal mine tunnel and the positive correlation between the phase envelope range and the wavelength, this paper proposes a structural design of the personnel positioning system of the coal mine tunnel based on the dual wavelength phase unwrapping, which combines the coherent detection structure with the dual wavelength phase measurement method in digital holography. In order to reduce false positives in the position information, the synthetic wavelength is used to increase the spread range of the phase. The experiment shows that this structure can reduce the false positioning without reducing the positioning accuracy, and the average double wavelength phase demodulation can reduce the positioning false alarm by 82.5%, which greatly improves the accuracy of the prediction of the tunnel construction safety, brings great protection to the personal and property safety of the tunnel construction personnel, and improves the mining efficiency of the coal mine.

## 1. Introduction

Coal is the main energy in the world. However, due to the characteristics of coal mine production, coal mine accidents occur from time to time. Therefore, it is very important to quickly resettle the trapped people and ensure the safe production of coal mines. As the underground production environment of coal mine is complex, it should mainly meet the requirements of dust-proof, explosion-proof, and intrinsically safe production of electrical and electronic equipment in coal mine [1, 2].

However, the existing personnel positioning systems, such as UWB (ultra wideband), super RFID, ZigBee, micro power, and Wi-Fi, have considerable background noise in the mine (mechanical noise of shearers, conveyors, crushers, and underground locomotives; plus electromagnetic noise generated by switch closing; and start and stop of large electromechanical equipment and operation of underground substations). Its application in the mine is limited by the power supply conditions at the site during emergency rescue operations [3–5]. The personnel positioning system based on optical fiber sensing and detection has attracted much attention because it is insensitive to electromagnetic interference and passive characteristics. In the error analysis of the existing phase difference positioning methods [6–8], the difference of the whole wavelength cycle is regarded as a fixed constant, that is, it is considered to have no impact on the ranging accuracy. However, recent studies have shown that there is a jump phenomenon in the difference of the whole number of wavelengths, and the influence of the phase difference measurement error must be considered when calculating the difference of the whole number of wavelengths using the phase difference measurement results [9, 10].

The existing fiber optic sensor positioning system is a distributed optical fiber personnel positioning system mainly based on the phase-sensitive optical time-domain reflectometry (Φ-OTDR) technology. Its main principle is the Rayleigh reflected light in the optical fiber phase changed by vibration, and the position of the vibration signal is calculated by the time delay between incident pulse light and backward Rayleigh scattering light [11]. As the relation between phase and time is a trigonometric function, the periodicity of which results in phase demodulation, the obtained vibration information may not necessarily be correct, and therefore, incorrect positioning occurs frequently.

Through the above research, it is found that the optical fiber sensor has greater advantages than other personnel safety positioning systems. Therefore, aiming at the shortcomings of the existing research and the characteristics of the existing mine personnel construction, this paper studies and designs a mine personnel positioning system based on the dual wavelength phase unwrapping method to reduce the positioning error caused by phase winding.

## 2. General Principle of the Optical Fiber-Based Positioning System in Mines

The working environment of the underground personnel in coal mines is special, the terrain structure of the mine is complex, the personnel and equipment are numerous, and the underground accidents are frequent. With the improvement of the state's emphasis on safety production and the promotion of safety production work, the positioning and safety monitoring of workers under the mine appear extremely important. Traditional underground safety positioning is simply based on radio frequency identification (RFID) technology to enable personnel to interact with RFID card reading equipment. However, this positioning method is limited by power consumption, equipment connection, signal environment, transmission distance, and transmission rate and cannot accurately locate each underground staff in real time. However, it is very difficult to design a complex optical fiber sensing system for mine positioning. In case of gas explosion, collapse, or flood, the rescue positioning system shall be directly connected to the existing optical fiber network to quickly locate the trapped personnel. However, the rescue team needs to carry all equipment except sensing fiber. Therefore, a simple but efficient coherent detection structure is more suitable for rescue operations in coal mine production environment. In addition, the coherent detection structure can effectively reduce noise interference [12–14].

**Figure 1:**
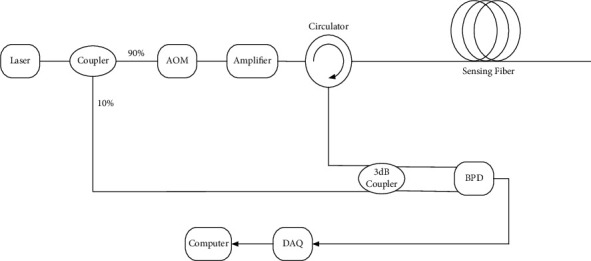
Coherent-detection structure for the mine personnel positioning system.

The typical structure of a personnel positioning system is shown in Figure 1. The underlying principle is that the incident light emitted by the laser is divided into two paths through the coupler—the reference light with 10% power and the detection light with 90% power. The detection light is modulated by the acousto-optic modulator (AOM), after enhancement of the power by the erbium-doped fiber amplifier (EDFA), and the detection light then enters the circulator and the sensing fiber. Rayleigh scattering light through the detection circulator enters the balance photoelectric detector (BPD) with the reference light. Finally, a high-speed data acquisition card (DAQ) is used to collect and demodulate vibration information in real time. The specific phase demodulation principle is described as follows.

The position of the disturbance point on the detection fiber can be determined by the phase difference between the detection light and the reference light, and its equation is given as follows:

(1) $$\begin{array}{c} ∆\varphi =\frac{4\pi n}{\lambda }x\text{.} \end{array}$$

In (1), Δ*φ*(*t*)is the phase difference between the detection light and reference light, *n* is the refractive index of the optical fiber, *λ* is the wavelength of the incident light, and *x* is the distance between the disturbance point on the detection fiber and the light source. According to (1), the specific disturbed position can be easily obtained as long as the phase difference is known. The specific phase demodulation steps are as follows.

**Figure 2:**
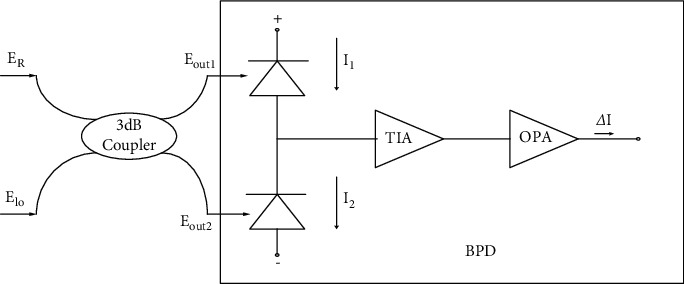
BPD working principle.

Firstly, the relationship between the phase difference and the intensity of Rayleigh reflected light and reference light is obtained through BPD. The process is shown in Figure 2. It is assumed that the optical fields input by the 3 dB coupler are Rayleigh reflected light *E*_R_ and reference light E_LO_, respectively, and its expression is as follows:

(2) $$\begin{array}{c} E_{R}=A\left(t\right)\exp\;\left[j\omega_{R}t+\varphi_{R}\left(t\right)\right]\text{,} \end{array}$$

(3) $$\begin{array}{c} E_{lo}=B\left(t\right)\exp\;\left[j\omega_{lo}t+\varphi_{lo}\left(t\right)\right]\text{.} \end{array}$$

In (2) and (3), A(t), *ω*_*R*_, and *φ*_R_(*t*) are the amplitude, frequency, and phase of Rayleigh reflected light, respectively; B(t),*ω*_lo_ and *φ*_lo_(*t*) are the amplitude, frequency, and phase of the reference light, respectively. The output Eout1 and Eout2 of the coupler through the two diodes of the BPD make a coherent heterodyne, respectively, and the output light field intensity is I_1_ and I_2_, respectively.

(4) $$\begin{array}{c} I_{1}\left(t\right)=\frac{k}{2}\left\{A^{2}\left(t\right)+B^{2}\left(t\right)+2A\left(t\right)B\left(t\right)\sin\;\left[\left(\omega_{R}-\omega_{lo}\right)t+\varphi_{R}\left(t\right)-\varphi_{lo}\left(t\right)\right]\right\}\text{,} \end{array}$$

(5) $$\begin{array}{c} I_{2}\left(t\right)=\frac{k}{2}\left\{A^{2}\left(t\right)+B^{2}\left(t\right)-2A\left(t\right)B\left(t\right)\sin\;\left[\left(\omega_{R}-\omega_{lo}\right)t+\varphi_{R}\left(t\right)-\varphi_{lo}\left(t\right)\right]\right\}\text{.} \end{array}$$

In (4) and (5), *k* is the responsivity of the detector, and the final output of BPD is as follows:

(6) $$\begin{array}{c} ∆I\left(t\right)=2kA\left(t\right)B\left(t\right)\sin\left[\left(\omega_{R}-\omega_{lo}\right)t+\varphi_{R}\left(t\right)-\varphi_{lo}\left(t\right)\right]\text{.} \end{array}$$

In (4) and (5), the meanings of symbols of all formulas are the same as above.

**Figure 3:**
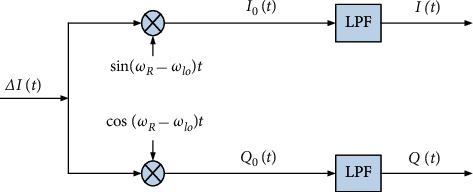
Quadrature phase demodulation.

It can be inferred from (6) that when the frequency shift of Rayleigh reflected light and reference light is not considered, the output of BPD is only related to *φ*_*R*_(*t*) − *φ*_lo_(*t*), that is, it is only related to the phase difference between Rayleigh reflected light and reference light, which is only recorded asΔ*φ*(*t*). For (6), the commonly used phase demodulation methods are Hilbert method, orthogonal phase method, and 3 × 3 optical fiber coupler method. The accuracy of Hilbert method is lower than that of the other two methods, and 3 × 3 optical fiber coupler method is more complex, and therefore, quadrature phase demodulation can be used. Its principle is shown in Figure 3.

Considering that Δ*I*(*t*) contains noise signals, (6) can be rewritten as follows:

(7) $$\begin{array}{c} \Delta I\left(t\right)=2kA\left(t\right)B\left(t\right)\sin\;\left[\left(\omega_{R}-\omega_{lo}\right)t+\Delta \varphi \left(t\right)\right]+n_{s}\left(t\right)\text{.} \end{array}$$

After the multiplier,

(8) $$\begin{array}{c} \begin{array}{c} I_{0}\left(t\right)=-kA\left(t\right)B\left(t\right)\left\{\cos\;\left[2\left(\omega_{R}-\omega_{lo}\right)+\Delta \varphi \left(t\right)\right]-\cos\;\left[\Delta \varphi \left(t\right)\right]\right\}+n_{s}\left(t\right)\sin\left(\omega_{R}-\omega_{lo}\right)t\\ Q_{0}\left(t\right)=kA\left(t\right)B\left(t\right)\left\{\sin\;\left[2\left(\omega_{R}-\omega_{lo}\right)+\Delta \varphi \left(t\right)\right]-\sin\;\left[\Delta \varphi \left(t\right)\right]\right\}+n_{s}\left(t\right)\cos\left(\omega_{R}-\omega_{lo}\right)t \end{array}\text{.} \end{array}$$

Subsequently, by passing it through the low-pass filter and removing the high-frequency component, the following equations are obtained:

(9) $$\begin{array}{c} I\left(t\right)=kA\left(t\right)B\left(t\right)\cos\;\left[\Delta \varphi \left(t\right)\right]\text{,} \end{array}$$

(10) $$\begin{array}{c} Q\left(t\right)=-kA\left(t\right)B\left(t\right)\sin\;\left[\Delta \varphi \left(t\right)\right]\text{.} \end{array}$$

The phase difference can be obtained from (9) and (10):

(11) $$\begin{array}{c} \Delta \varphi \left(t\right)=-\arctan\frac{Q\left(t\right)}{I\left(t\right)}\text{.} \end{array}$$

**Figure 4:**
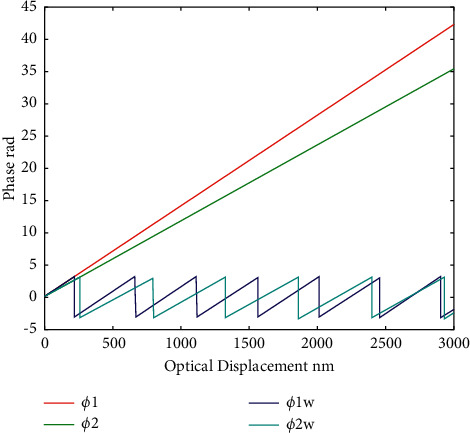
Wrapping of the phase.

It can be observed that the interference of noise in the system can be effectively filtered out through orthogonal demodulation. Due to the periodicity of trigonometric function, the value range of tangent function in (11) is (−*π*/2, *π*/2) that can be expanded to (−*π*, *π*) through the positive and negative signs of I(t) and Q(t). However, the phase demodulation is still extremely limited. When the measurement information significantly changes and the obtained phase information exceeds the range of (−*π*, *π*), phase winding occurs, and the obtained phase information is no longer the real phase. Refer to Figure 4 for details.

In Figure 4, *φ*_1*w*_ and *φ*_2*w*_ are the winding phases of 1310 and 1550 nm wavelengths, respectively. *φ*_1_ is the phase distribution of *φ*_1*w*_ after unwrapping, and *φ*_2_ is the phase distribution of *φ*_2*w*_ after unwinding. Due to the periodicality of the phase trigonometric function, the phase jumps every time when it passes through an integer multiple of *π*. In traditional phase unwrapping, the relationship between the winding phase and the principal phase is as follows. For the comparison of two phase values, when phase jump occurs, if the phase difference of two adjacent values is more than 2*π*, after that point, each point of the phase values is ±2*π* and to include the difference between adjacent sampling point within (−*π*, *π*), but the method of adjacent between two points after the final compensation difference is more than 2*π* or −2*π*. Then, the traditional phase unwinding method will be invalid. As can be observed from Figure 4, the time interval for the generation of jump increases when a laser source with a relatively long wavelength is used. Therefore, the existing fiber laser source can be used to avoid the aforementioned problems and obtain accurate phase distribution through dual-wavelength phase demodulation method in digital holography [15–18].

## 3. Structure and Phase Demodulation Principle of the Dual Wavelength Method in the Personnel Positioning System

The structure of the improved mine personnel positioning system is similar to the coherent detection structure, except that the optical signals of 1310 and 1550 nm wavelengths are coupled through the coupler and enter the detection arm and reference arm simultaneously. Before entering the 3 dB coupler, the wave is divided through a demultiplexer, so that the phase change of the disturbance point for the three wavelengths can be obtained. The positioning misjudgment caused by phase jump can be corrected according to the other two phase changes simultaneously.

**Figure 5:**
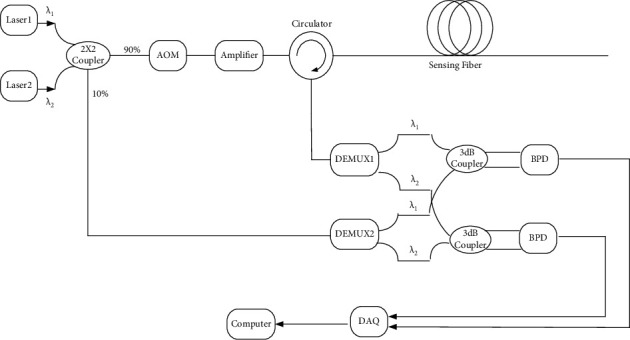
Structure of dual wavelength method for positioning system in mines.

The dual wavelength phase coherent demodulation structure is shown in Figure 5, and the synthetic wavelength of the system is as follows:

(12) $$\begin{array}{c} \lambda_{s}=\frac{\lambda_{1}\lambda_{2}}{\left|\lambda_{1}-\lambda_{2}\right|}\text{.} \end{array}$$

The modulation mode in which the deviation value of the carrier phase from its reference phase varies in proportion to the instantaneous value of the modulation signal is called phase modulation. Phase modulation and frequency modulation are closely related. During phase modulation, frequency modulation occurs simultaneously. During frequency modulation, phase modulation also occurs simultaneously, but the change laws of the two are different.

*λ*s, *λ*_1_, and *λ*_2_ are the wavelengths of the synthesized optical signal, light source 1, and light source 2, respectively. The wave length thus synthesized is greater than the wavelength of light source 1 or light source 2. The specific phase demodulation steps are as follows:

1. Wavelengths *λ*1 and *λ*2 are derived from the demultiplexers DEMUX1 and DEMUX2, respectively. The corresponding uncompensated wrapping phases *φ*_1W_(*t*) and *φ*_2W_(*t*) are obtained by BPD. The winding phase of synthetic wavelength is obtained through *φ*_1*W*_(*t*) − *φ*_2*W*_(*t*).
2. Zero crossing compensation is performed for the wrapping phase *φ*_*sw*_(*t*)of the synthetic wavelength. When the value of the wrapping phase is less than zero, 2*π* is added to it, and then the adjacent two points are compared. When the difference exceeds *π*, the phase is unwrapped. Finally, the synthetic phase unwrapping result *φ*_*s*_(*t*) is obtained.
3. The wrapping phases of wavelength1 *φ*_1W_(*t*) and wavelength2 *φ*_2W_(*t*) are unwrapped to obtain phase unwrapping results *φ*_1_(*t*) and *φ*_2_(*t*).
4. When the system is positioning, the false position information is eliminated by the synthetic phase unwrapping result, and then through the wavelength *λ*1 or wavelength *λ*2 to determine the accurate positioning distance and improve the positioning accuracy.

## 4. Experiment and Discussion

To verify the performance of dual-wavelength mine personnel positioning system, relevant experiments are designed. Using cylindrical piezoelectric ceramics (PZT) as the external vibration source, according to the References [19, 20], a significant gap occurs in the vibration range caused by knocking. The vibration range of tunnel's surrounding rock will not exceed 10 m, except that the surrounding rock and metal objects such as anchor bolts placed in the surrounding rock of the mine tunnel will have a vibration range of more than 20 m. Therefore, in the experiment, the length of the optical cable winding PZT is 3 m, which is used to simulate the vibration of underground personnel knocking on the mine wall. PZT was placed at a position of 1100 m on the optical fiber detection arm. The light sources were laser diodes with wavelengths of 1310 and 1550 nm, and their spectral linewidth is within 10 nm when the output power is at 20∼50 mW. Theoretically, the closer the wavelengths of the two lasers, the longer the synthetic wavelength, and the less likely it is to produce phase winding. However, limited by spectral linewidth, unnecessary errors occur when the wavelengths of the two lasers are too close.

**Figure 6:**
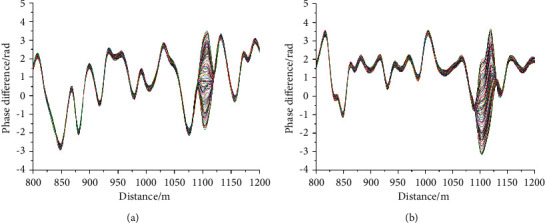
Phase demodulation results. (a) 100 phase difference curve at 1100 m with single wavelength demodulation (b) 100 phase difference curve at 1100 m with dual wavelength demodulation.

The positioning results are shown in Figure 6. The a and *b* diagrams of Figure 6 show the positioning diagrams of single wavelength of 1310 nm and dual wavelength phase demodulation, respectively. The personnel position information is obtained by using 100 phase difference curves of the vibration position. Comparing the two methods, it can be observed that due to the use of orthogonal demodulation, the noise of the two positioning methods is small and the curve is smooth. The disturbance range of single wavelength positioning is smaller than that of dual wavelength positioning. Therefore, single wavelength has certain advantages in using disturbance peak for accurate positioning.

The location of both single wavelength and dual wavelength lags behind the actual PZT disturbance. On analysis, it was found that the lag is caused by the low-pass digital filter in the coherent demodulation and can be determined by the order of the low-pass digital filter. The equation is as follows:

(13) $$\begin{array}{c} L_{r}=\frac{N_{0}c}{4f_{s}n}\text{.} \end{array}$$

**Figure 7:**
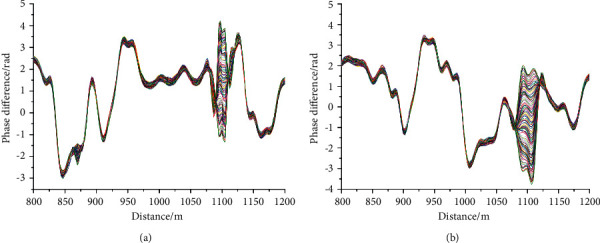
Corrected positioning results. (a) Corrected single wavelength phase demodulation (b) corrected dual-wavelength phase demodulation.

In (13), *L*_*r*_ is the lag distance, *N*_0_ is the order of the digital filter, and *f*_*s*_ is the sampling frequency of DAQ. The positioning result through delay compensation is shown in Figure 7, wherein the corrected single wavelength phase demodulation is shown in Figure 7(a) and the corrected dual wavelength phase demodulation is shown in Figure 7(b). It can be observed from Figure 7 that the positioning delay of single wavelength and dual wavelength has been significantly improved compared with Figure 6, but it has no impact on the interference range of the two demodulation methods.

**Figure 8:**
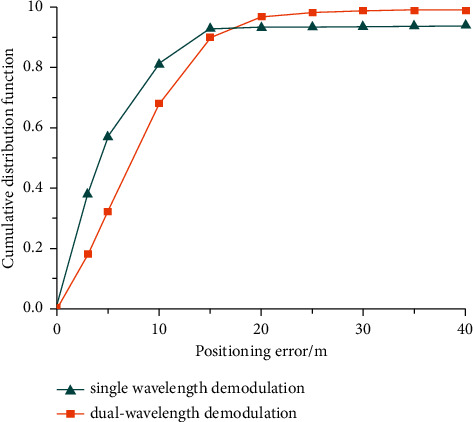
Positioning error accumulation curve of two-phase demodulation methods.

The error accumulation curves of the two positioning methods for several phase demodulation positioning iterations are shown in Figure 8.

Figure 8 shows the cumulative distribution curve of the positioning error after multiphase demodulation. It can be observed that the positioning error is within 14 meters, but the error of dual wavelength is more than 14 meters, and the maximum error value is close to 30 meters. It can be seen that single wavelength phase demodulation has significant advantages, while the positioning information error of dual wavelength demodulation is large. However, beyond the positioning error range of 17 meters, dual wavelength phase demodulation can significantly eliminate incorrect positioning. The result of single wavelength demodulation is equivalent to the positioning accuracy described in document [21]. Therefore, it can be inferred from the curve in Figure 8 that when the positioning error is greater than 14 m, the cumulative error of the single wavelength phase demodulation system will not increase and will eventually stabilize at about 94%. The cumulative error of the dual wavelength phase demodulation system is stable at 98% when the positioning error is 20 m and 99% when the positioning error is 30 m.

The specific phase errors of the two demodulation methods are listed in Table 1. It can be observed that as PZT is wound with a 3 m optical fiber to simulate the knocking vibration of the mine wall, the accuracy of the two demodulation methods with positioning error within 3 m is relatively small, especially when the positioning accuracy of dual wavelength phase demodulation is only 18.24%. When the positioning error is within 10 m, it can be observed that single wavelength phase demodulation has significant advantages, and its positioning accuracy is 81.22%. When the positioning error is required to be within 20 m, the accuracy of the two demodulation methods is close, that is, the single wavelength demodulation method is 93.18%, and the dual wavelength demodulation method is 96.74%. When the positioning error is required to be within 30 m, it can be observed that the accuracy of dual wavelength positioning is significantly higher than that of single wavelength, and at this time, the accuracy of dual wavelength positioning reaches 98.88%. When the positioning error is more than 30 m, the positioning information can be regarded as incorrect information. At this time, the incorrect rate of single wavelength positioning is 6.41%, while that of dual wavelength positioning is only 1.12%. Therefore, it can be concluded that dual wavelength phase demodulation can reduce positioning false positives by 82.5%. Due to the addition of wave splitter, coupler, and photoelectric balancer, the positioning time of dual wavelength positioning system is slightly lower than that of single wavelength positioning system, but in practical engineering application, such time difference will not affect the actual application.

Therefore, when using the dual wavelength phase demodulation system for mine personnel positioning, the positioning results can be used to determine the approximate positioning information, and then the approximately similar single wavelength positioning can be selected through computer comparison to eliminate other false alarm information. The positioning distance of the compared single wavelength positioning system can be considered the final positioning information. If the two single wavelength positioning results are more than 30 m different from the positioning results of the synthetic wavelength, the positioning results of the synthetic wavelength are considered the final positioning information.

## 5. Conclusion

Coal mine roadway personnel positioning system has always been an important guarantee for coal mine safety production. A simple but reliable positioning system is needed for personnel positioning and rescue operations in coal mine tunnels. General coherent detection systems often misreport positioning information due to phase winding, thus wasting valuable initial rescue time. Therefore, this paper presents a structural design of a personnel positioning system for coal mine tunnel based on dual wavelength phase unwrapping, which combines the coherent detection structure with the dual wavelength phase measurement method in digital holography. In order to reduce false positives in the position information, the synthetic wavelength is used to increase the spread range of the phase and the following conclusions were drawn:

1. The dual wavelength mine personnel positioning system uses the positive correlation between the phase envelope and the wavelength to expand the phase range by using a longer synthetic wavelength. By cooperating with single wavelength phase demodulation, false positives can be reduced under the condition of satisfying positioning accuracy.
2. It is found that the mine personnel positioning system based on dual wavelength phase unwrapping proposed in this paper can greatly improve the positioning accuracy of mine personnel and greatly reduce the probability of positioning false alarm. Its maximum phase demodulation can reduce the positioning false alarm by 82.5%.
3. Compared with the original coherent structure, the system constructed in this paper only adds a small number of couplers, BPD, DEMUX, and other components, which proves that the mine personnel positioning system constructed in this paper based on dual wavelength phase expansion can meet the simple and reliable requirements of engineering practice.

In conclusion, it can be seen that the main optical fiber detection arm still adopts the process of laying optical fiber in the original mine, and the self-contained equipment is less, which can fully meet the needs of emergency rescue for underground accidents in coal mines.

## Figures and Tables

**Table 1:** Error comparison of two-phase demodulation methods.

Demodulation method	Proportion of positioning error <3 m (%)	Proportion of positioning error <10 m (%)	Proportion of positioning error <20 m (%)	Proportion of positioning error <30 m (%)	Average positioning time (s)
Single wavelength phase demodulation	38.12	81.22	93.18	93.59	2.56
Synthetic wavelength phase demodulation	18.24	68.27	96.74	98.88	3.24

## Data Availability

The datasets generated during and/or analyzed during the current study are not publicly available due to sensitivity and data use agreement.
